# Translesion-synthesis-mediated bypass of DNA lesions occurs predominantly behind replication forks restarted by PrimPol

**DOI:** 10.1016/j.celrep.2025.115360

**Published:** 2025-02-26

**Authors:** Ashna Dhoonmoon, Julia R. Ambrose, Sonal Garg, Cynthia Lascarez-Espana, Abbey Rebok, Thomas E. Spratt, George-Lucian Moldovan, Claudia M. Nicolae

**Affiliations:** 1 Department of Biochemistry and Molecular Biology, The Pennsylvania State University College of Medicine, Hershey, PA 17033, USA; 2 These authors contributed equally; 3 Lead contact

## Abstract

The bypass of DNA lesions by translesion synthesis (TLS) polymerases is a critical step for DNA damage tolerance, allowing the completion of DNA synthesis. It has been under debate whether TLS-mediated bypass restarts stalled forks or occurs post-replicationally. We developed cell imaging techniques based on proximity ligation to monitor the recruitment of TLS polymerases Polκ and Polη to DNA adducts. We show that this recruitment is adduct specific, with Polκ being preferentially recruited to benzo[*a*]pyrene diol epoxide (BPDE) lesions and Polη to cisplatin lesions. The recruitment depends on the primase-polymerase PrimPol, which reprimes downstream of stalled forks to restart DNA synthesis. TLS polymerase deficiency results in the accumulation of single-stranded DNA (ssDNA) gaps in an adduct-specific manner, which are processed into double-strand breaks (DSBs). Our findings argue that TLS occurs mainly behind the restarted replication fork in order to fill PrimPol-derived gaps and is essential to suppress the nucleolytic conversion of ssDNA gaps into cytotoxic DSBs in a lesion-specific manner.

## In brief

Dhoonmoon et al. investigate the lesion-specific recruitment of translesion synthesis polymerases during DNA replication stress. They show that translesion synthesis occurs predominantly behind the restarted replication fork in order to fill PrimPol-derived gaps. This is essential to suppress the nucleolytic conversion of ssDNA gaps into cytotoxic DSBs.

## Graphical Abstract

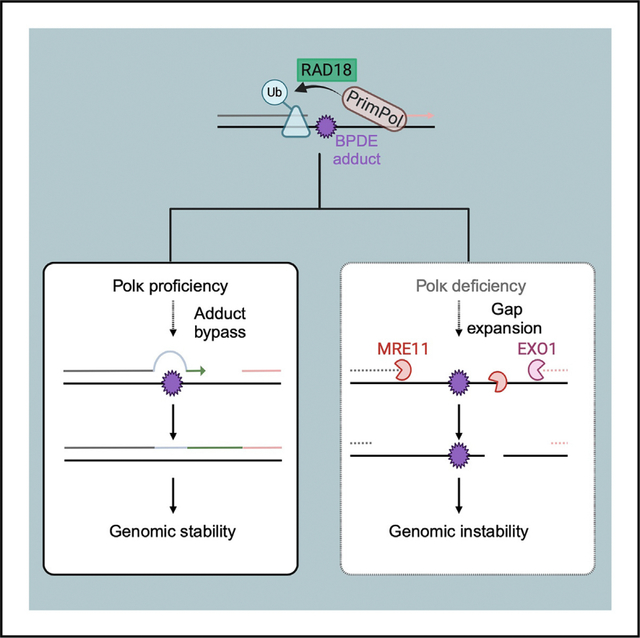

## INTRODUCTION

DNA adducts, predominantly affecting only one strand of the DNA, can be induced by a variety of DNA-damaging agents.^1–5^ Formation of these adducts is relevant for both cancer etiology and cancer treatment. Benzo[*a*]pyrene diol epoxide (BPDE)-DNA adducts derived from exposure to benzo[*a*]pyrene, a chemical found in tobacco smoke and other combustion fumes, have been associated with lung carcinogenesis. These adducts cannot be accurately replicated by DNA polymerases during S phase, causing point mutagenesis. On the other hand, cisplatin-DNA adduct formation represents the main mechanism of action of cisplatin chemotherapy, overwhelming the DNA repair machinery.

DNA replication predominately involves high-fidelity polymerases (Polε and Polδ), which are arrested upon encountering modified bases on the template strand. A major cellular mechanism handling the cellular tolerance to DNA adducts is translesion synthesis (TLS). TLS involves specialized polymerases able to replicate through DNA lesions.^6^ By inserting nucleotides across DNA adducts, these polymerases cause increased mutagenesis.

Biochemical studies using recombinant proteins and DNA substrates with various adducts have shown that DNA adducts can be bypassed by multiple polymerases but with different efficacies. Cisplatin-DNA adducts can be most efficiently replicated by Polη and less so by Polκ.^7,8^ In contrast, Polκ bypasses BPDE adducts in a largely error-free manner, while Polη bypasses these adducts in a mutagenic manner.^9–13^ These results were further confirmed by studies using Polκ-deficient cells, which accumulated BPDE-induced mutations.^11,12,14^ Moreover, both Polκ and Polη were shown to form BPDE-induced foci using fluorescently tagged, exogenously expressed polymerase variants.^15^ However, loss of Polκ, but not Polη, resulted in increased γH2AX phosphorylation upon BPDE exposure. Based on these findings, it was proposed that in BPDE-treated Polκ-deficient cells, arrested forks collapse to generate double-strand breaks (DSBs).

To avoid unnecessary mutagenesis, TLS polymerases are kept under tight cellular control. The main regulatory mechanism is thought to be the mono-ubiquitination of the polymerase co-factor PCNA, a ring-shaped homotrimeric protein that encircles DNA at replication forks and binds DNA polymerases, conferring their processivity.^16^ In response to DNA damage, PCNA gets mono-ubiquitinated on chromatin. Since TLS polymerases have higher affinity for ubiquitinated PCNA, they may replace the replicative polymerases upon PCNA mono-ubiquitination.

Although the exact timing and location of PCNA ubiquitination in cells were only poorly defined, the discovery of PCNA ubiquitination lent support to the “on-the-fly” model,^17^ which posits that TLS polymerases are recruited to stalled replication forks to restart them by bypassing the DNA lesion. Following this, PCNA is deubiquitinated to allow the switch back to replicative polymerases.

In higher eukaryotes, stalled forks can also be restarted through the activity of PrimPol, which has both primase and polymerase activities.^18,19^ PrimPol-mediated restart leaves behind single-stranded DNA (ssDNA) gaps to be filled in later. Gap filling can occur through a BRCA pathway-dependent process, possibly involving recombination with the nascent strand of the sister chromatid.^20^ Separately, TLS has also been proposed to be involved in the filling of PrimPol-mediated gaps.^21^ PrimPol-mediated repriming was found in response to BPDE^22^ and cisplatin.^23^ Moreover, BRCA-deficient cells are hypersensitive to TLS inhibition, which led to the model that TLS fills gaps in these cells.^24,25^

While the overall relevance of TLS for adduct tolerance is clear, to what extent the bypass of specific adducts is dependent on specific polymerases in cells is unclear, as most of the specificity studies were performed in biochemical systems using recombinant proteins, complemented by genetic mutagenesis studies. The lack of cell-based approaches to measure TLS polymerase engagement also makes it difficult to assess to what extent TLS polymerases are involved in restarting arrested replication forks (on the fly) compared to gap filling.

To address these issues, we developed proximity-ligation-based imaging methods to detect the recruitment of endogenous TLS polymerases to nascent DNA upon adduct generation by BPDE and cisplatin. We show that TLS polymerases are recruited to adducts in a specific manner, with Polκ predominantly recruited to BPDE adducts and Polη predominantly recruited to cisplatin adducts. Moreover, we show that this recruitment, as well as PCNA ubiquitination upon adduct formation, depends on PrimPol, indicating that TLS predominantly occurs during gap filling rather than on the fly. In line with this, we find that loss of TLS polymerases results in adduct-induced ssDNA gap accumulation in an adduct- and polymerase-specific manner. These gaps are subsequently processed by DNA nucleases, which convert them into double-strand DNA breaks (DSBs). Finally, we show that this conversion underlies the cytotoxicity of DNA adduct-inducing agents.

## RESULTS

### Specific recruitment of TLS polymerases Polκ and Polη to BPDE and cisplatin adducts during DNA synthesis in human cells

**Figure 1.**
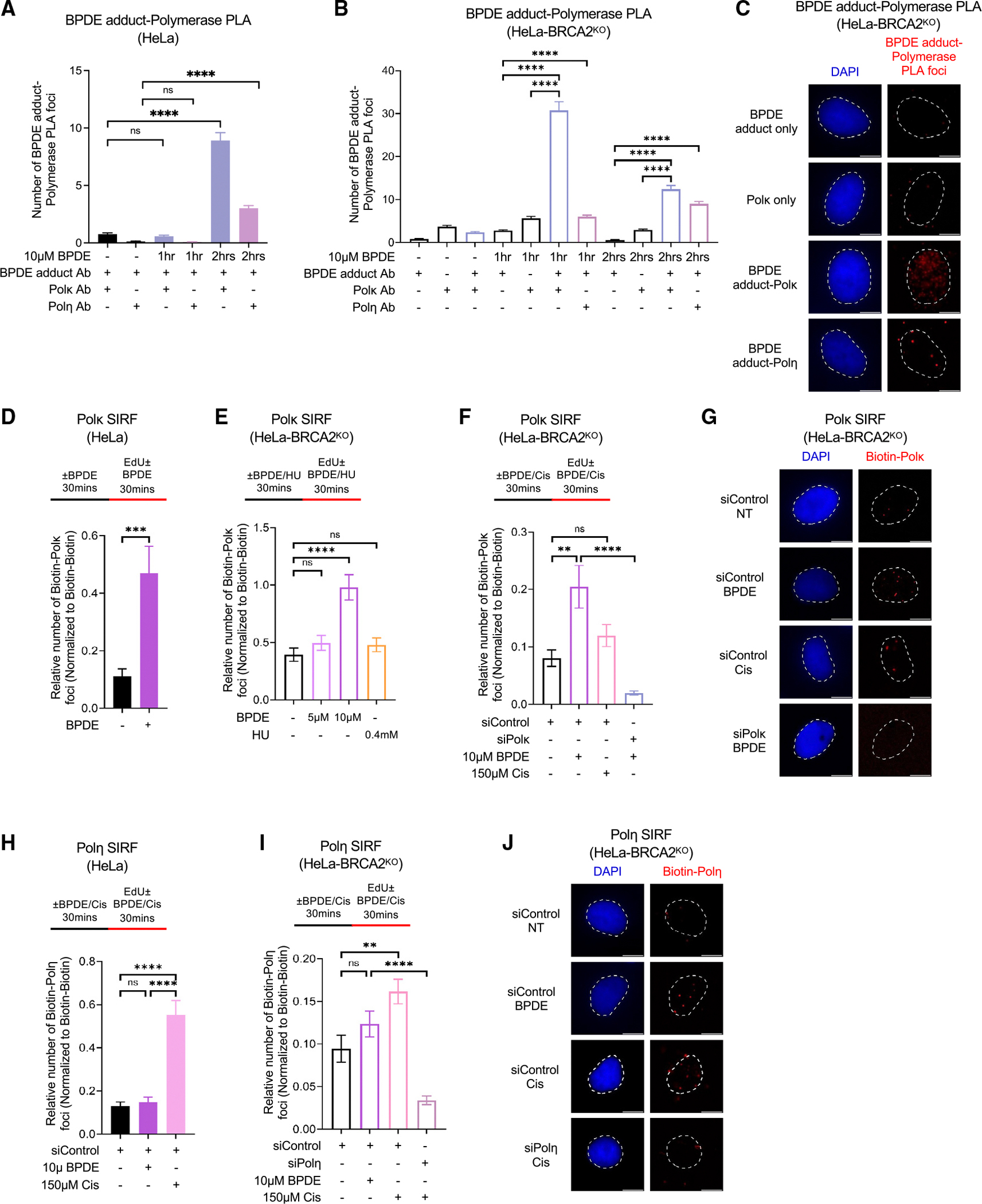
Adduct-specific recruitment of TLS polymerases to nascent DNA (A–C) Proximity ligation assay showing the specific colocalization of Polκ or Polη with BPDE adducts in wild-type and BRCA2-knockout (BRCA2^KO^) (B and C) HeLa cells. Single antibody controls are used to confirm the specificity of the PLA signals observed. Quantifications (A and B) and representative micrographs (C), with scale bars representing 10 μm, are shown. At least 75 cells were quantified for each condition. Bars indicate the mean values, error bars represent standard errors of the mean, and asterisks indicate statistical significance (t test, two-tailed, unpaired). (D–G) SIRF experiments showing the specific recruitment of Polκ to nascent DNA upon treatment with BPDE in HeLa cells. Knockdown of Polκ is used as control to confirm the specificity of the SIRF signals observed. Quantifications (D–F) and representative micrographs (G), with scale bars representing 10 μm, are shown. At least 75 cells were quantified for each condition. Bars indicate the mean values, error bars represent standard errors of the mean, and asterisks indicate statistical significance (t test, two-tailed, unpaired). Schematic representations of the assay conditions are shown at the top. Western blots confirming Polκ knockdown are shown in Figure S1A. (H–J) SIRF experiments showing the recruitment of Polη to nascent DNA upon treatment with BPDE or cisplatin in HeLa cells. Knockdown of Polη is used as control to confirm the specificity of the SIRF signals observed. Quantifications (H and I) and representative micrographs (J), with scale bars representing 10 μm, are shown. At least 75 cells were quantified for each condition. Bars indicate the mean values, error bars represent standard errors of the mean, and asterisks indicate statistical significance (t test, two-tailed, unpaired). Schematic representations of the assay conditions are shown at the top. Western blots confirming Polη knockdown are shown in Figure S1B. See also Figure S1 and Table S1.

The biochemical and genetic studies described above have indicated that both Polκ and Polη can bypass BPDE adducts, with Polκ specifically being the polymerase required to suppress BPDE-induced mutagenesis. The mechanism of this bypass in cells is still unclear. A specific antibody recognizing BPDE-DNA adducts has been developed to quantify these adducts in cells. We sought to employ this antibody in proximity ligation assays (PLAs) to investigate the timing and regulation of endogenous TLS polymerase recruitment to BPDE adducts in HeLa cells. We performed these experiments in both wild-type cells and BRCA2-knockout cells, which are defective in homologous recombination (HR)-mediated mechanisms of fork protection and may thus be more reliant on TLS, allowing us to detect more subtle changes in PLA signals. In wild-type cells, we detected colocalization of endogenous Polκ with BPDE adducts after 2 h of treatment with 10 μM BPDE, while the colocalization of Polη with BPDE adducts was also detected under these conditions but at lower levels (Figure 1A). In BRCA2-knockout cells, we observed that endogenous Polκ colocalizes with BPDE adducts after 1 h of treatment with 10 μM BPDE, while Polη-BPDE PLA foci were not detected under these conditions (Figures 1B and 1C). Instead, Polη was found to colocalize with BPDE adducts after 2 h of treatment but at lower levels than Polκ. Overall, these findings suggest that the Polκ interaction with BPDE adducts occurs earlier and at higher levels than that of Polη, confirming its specificity for handling these adducts in cells.

While the PLAs allowed us to detect the interaction of specific TLS polymerases with BPDE adducts in cells, they cannot inform on the timing of these interactions with respect to DNA replication. To address this, we turned to PLA-based SIRF (quantitative *in situ* analysis of protein interactions at DNA replication forks) assays.^26^ SIRF assays allow the quantification of protein binding to nascent DNA upon labeling of nascent strands using EdU incorporation and the quantification of endogenous protein recruitment to EdU-labeled DNA using PLA. In both wild-type and BRCA2-knockout HeLa cells, treatment with 10 μM BPDE for 1 h (with EdU labeling during the final 30 min of the treatment) resulted in a specific increase in Polκ recruitment to EdU-labeled nascent DNA (Figures 1D–1G), indicating that Polκ is recruited to BPDE-DNA adducts on the parental strand during DNA synthesis. As controls, cisplatin treatment did not cause an increase in Polκ SIRF foci, while Polκ depletion reduced this signal (Figures 1F, 1G, and S1A). In contrast, we observed a specific recruitment of Polη to nascent DNA upon cisplatin treatment (Figures 1H–1J). As control, Polη depletion reduced the Polη SIRF signal as expected (Figures 1I, 1J, and S1B). In conclusion, by developing specific imaging methods to quantify interactions of endogenous TLS polymerases with DNA adducts during DNA synthesis in cells, we found that Polκ is specifically recruited to nascent DNA upon BPDE-DNA adduct formation, while Polη is specifically recruited to cisplatin-DNA adducts under these conditions.

### PCNA ubiquitination on nascent DNA and TLS polymerase recruitment depend on PrimPol

**Figure 2.**
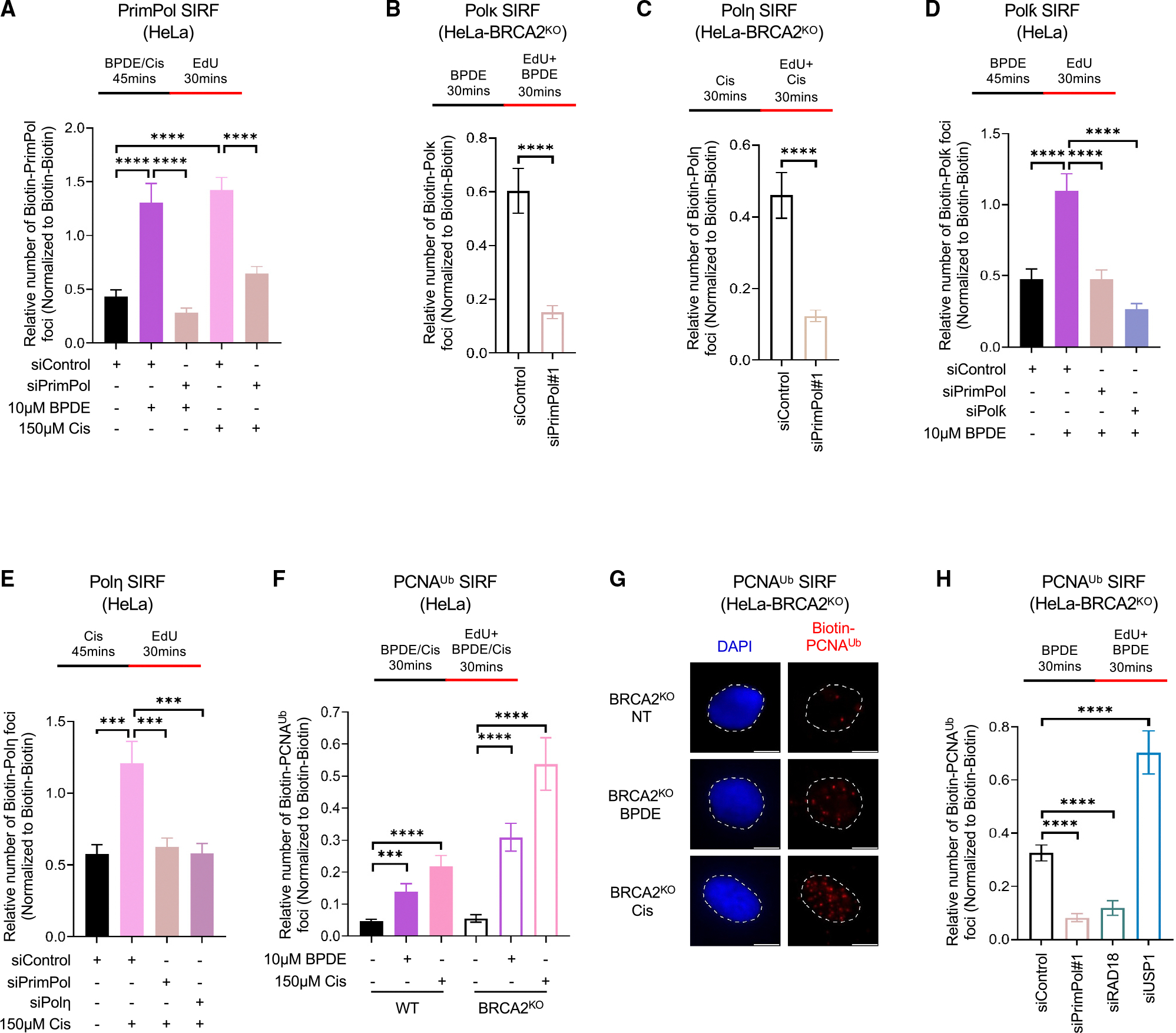
PCNA ubiquitination at stalled forks and TLS polymerase recruitment require PrimPol-mediated restart (A) SIRF assays showing the recruitment of PrimPol to nascent DNA upon treatment with BPDE or cisplatin in HeLa cells. Knockdown of PrimPol is used as control to confirm the specificity of the SIRF signals observed. At least 80 cells were quantified for each condition. Bars indicate the mean values, error bars represent standard errors of the mean, and asterisks indicate statistical significance (t test, two-tailed, unpaired). A schematic representation of the assay conditions is shown at the top. (B–E) SIRF assays showing that depletion of PrimPol suppresses the recruitment of TLS polymerases Polκ (B and D) and Polη (C and E) to nascent DNA upon adduct formation in HeLa cells. At least 75 cells were quantified for each condition. Bars indicate the mean values, error bars represent standard errors of the mean, and asterisks indicate statistical significance (t test, two-tailed, unpaired). Schematic representations of the assay conditions are shown at the top. Western blots confirming PrimPol knockdown are shown in Figure S1C. (F–H) SIRF experiments showing that PCNA ubiquitination on nascent DNA upon adduct induction requires PrimPol in HeLa cells. Quantifications (F and H) and representative micrographs (G), with scale bars representing 10 μm, are shown. Knockdowns of RAD18 and USP1 were used as controls. At least 75 cells were quantified for each condition. Bars indicate the mean values, error bars represent standard errors of the mean, and asterisks indicate statistical significance (t test, two-tailed, unpaired). Schematic representations of the assay conditions are shown at the top. Western blots confirming RAD18 and USP1 knockdowns are shown in Figures S1D and S1E. See also Figures S1–S3 and Table S1.

Having established these specific imaging tools, we next employed them to test if TLS is involved in the restart of stalled replication forks arrested at DNA adducts (on-the-fly TLS) or in filling ssDNA gaps resulting from fork restart downstream of the lesion. The main mechanism of fork restart downstream of DNA lesions in mammalian cells involves the primase-polymerase PrimPol. Indeed, PrimPol SIRF assays showed that endogenous PrimPol is recruited to nascent DNA under the BPDE or cisplatin treatment conditions investigated above for Polκ and Polη (Figure 2A). We thus sought to investigate the impact of PrimPol loss on TLS polymerase recruitment to nascent DNA upon exposure to adduct-inducing DNA-damaging agents. In both wild-type and BRCA2-knockout cells, depletion of PrimPol, using two different small interfering RNA (siRNA) oligonucleotides, significantly reduced the recruitment of Polκ to nascent DNA upon BPDE treatment, as well as the recruitment of Polη to nascent DNA upon cisplatin treatment, without affecting EdU incorporation levels (Figures 2B, 2C, S1C, S2A–S2D, S3A, and S3B). Moreover, we confirmed these findings using a different SIRF labeling scheme in which the nascent strand was EdU labeled only after the removal of the adduct-inducing agents. Under these labeling conditions, we also observed a specific binding of Polκ and Polη to nascent DNA in wild-type HeLa cells upon BPDE and cisplatin treatment, respectively, which was suppressed by PrimPol depletion (Figures 2D and 2E). These findings suggest that Polκ and Polη are predominantly recruited to DNA adducts upon fork restart downstream of the lesion by PrimPol.

Previous studies, using western blot assays, showed that PCNA is ubiquitinated upon BPDE and cisplatin treatments,^27,28^ and co-immunoprecipitation interactions indicated that both Polκ and Polη interact preferentially with the mono-ubiquitinated form of PCNA.^28,29^ Thus, PCNA ubiquitination at stalled replication forks may represent the trigger that recruits Polκ to BPDE adducts and Polη to cisplatin adducts. We thus sought to test if we can detect PCNA ubiquitination on nascent DNA using SIRF experiments under the conditions used to detect Polκ and Polη recruitment in the experiments described above. Using an antibody specifically detecting ubiquitinated PCNA, we found that both BPDE and cisplatin treatments resulted in PCNA ubiquitination on nascent DNA (Figures 2F and 2G). As controls, depletion of the PCNA ubiquitin ligase RAD18 reduced ubiquitinated PCNA SIRF foci upon BPDE exposure, while depletion of the PCNA deubiquitinating enzyme USP1 increased these foci (Figures 2H, S1D, S1E, and S3C). Moreover, depletion of the ubiquitin-conjugating enzyme UBC13, responsible for PCNA polyubiquitination, did not reduce the ubiquitinated PCNA SIRF signal under the BPDE and cisplatin treatment conditions employed (Figures S1F and S2E), indicating that the signal derives from mono-ubiquitinated PCNA.

We next investigated the impact of PrimPol loss on PCNA ubiquitination on nascent DNA. Using two different PrimPol siRNA oligonucleotides, we found that PrimPol depletion suppressed both the BPDE-induced and cisplatin-induced PCNA ubiquitination SIRF signals to similar levels as RAD18 depletion (Figures 2H and S2F–S2H). These findings suggest that the ubiquitination of PCNA occurs upon restart of stalled replication forks by PrimPol downstream of the lesion.

**Figure 3.**
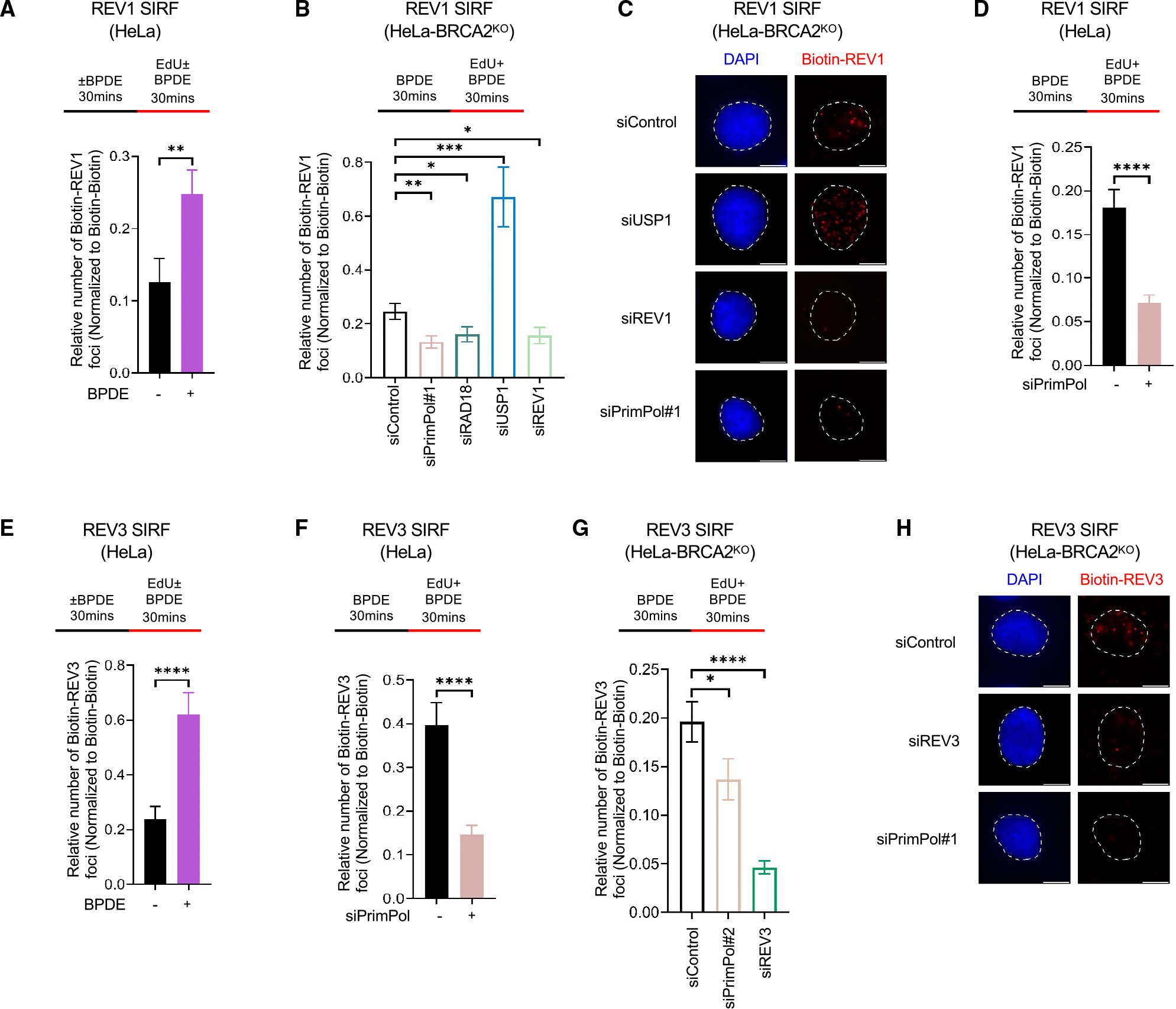
Recruitment of extension polymerases REV1 and REV3 to BPDE adducts on nascent DNA is PrimPol dependent (A–D) SIRF experiments showing that PrimPol depletion reduces the recruitment of REV1 to nascent DNA upon treatment with BPDE in HeLa cells. Knockdown of REV1 is used as control to confirm the specificity of the SIRF signals observed. Quantifications (A, B, and D) and representative micrographs (C), with scale bars representing 10 μm, are shown. At least 75 cells were quantified for each condition. Bars indicate the mean values, error bars represent standard errors of the mean, and asterisks indicate statistical significance (t test, two-tailed, unpaired). Schematic representations of the assay conditions are shown at the top. Western blots confirming REV1 knockdown are shown in Figure S1G. (E–H) SIRF experiments showing that PrimPol depletion reduces the recruitment of REV3 to nascent DNA upon treatment with BPDE in HeLa-BRCA2^KO^ cells. Knockdown of REV3 is used as control to confirm the specificity of the SIRF signals observed. Quantifications (E–G) and representative micrographs (H), with scale bars representing 10 μm, are shown. At least 70 cells were quantified for each condition. Bars indicate the mean values, error bars represent standard errors of the mean, and asterisks indicate statistical significance (t test, two-tailed, unpaired). Schematic representations of the assay conditions are shown at the top. See also Figures S1–S3 and Table S1.

Bypass of DNA lesions by TLS is thought to entail a multi-step process, with the initial bypass polymerase inserting nucleotides across the lesion and the polymerase Polζ extending these nucleotides over a longer stretch.^30–33^ The switch between the bypass and extension polymerases is mediated by REV1, which interacts with both polymerases, providing a platform for this process.^34–36^ We thus sought to investigate if the recruitment of the extension complex to nascent DNA upon fork adduct formation is also dependent on PrimPol. Using SIRF experiments, we found that, in both wild-type and BRCA2-knockout cells, REV1 is recruited to nascent DNA upon BPDE exposure (Figures 3A–3C). As a control, REV1 depletion reduced the REV1 SIRF signal as expected (Figures 3B, 3C, and S1G). Moreover, this signal was increased by USP1 depletion and decreased upon RAD18 depletion, indicating the role of PCNA ubiquitination in this process. Depletion of PrimPol, with two different siRNA oligonucleotides, also reduced this signal to the same extent (Figures 3B–3D, S2I, and S3D), indicating that PrimPol is required for the recruitment of REV1 to the nascent strand upon BPDE adduct formation.

Finally, we also investigated the recruitment of Polζ. Polζ is composed of the catalytic subunit REV3 and the regulatory subunits REV7, POLD2, and POLD3. We focused our analyses on REV3. Using SIRF assays, we were able to detect nascent strand recruitment of REV3 upon BPDE exposure (Figures 3E–3H). As a control, REV3 depletion reduced the REV3 SIRF signal as expected (Figures 3G and 3H). A similar dependency on PCNA ubiquitination, as in the case of REV1, was observed for REV3 (Figure S2J). Depletion of PrimPol, with two different siRNA oligonucleotides, also reduced this signal to the same extent (Figures 3F–3H, S2K, and S3E), indicating that PrimPol is also required for the recruitment of Polζ to the nascent strand upon BPDE adduct formation.

Overall, these findings indicate that both PCNA ubiquitination and the recruitment of bypass and extension TLS polymerases predominantly occur behind replication forks restarted by PrimPol downstream of the arresting DNA adduct.

### TLS polymerases fill ssDNA gaps induced by DNA adducts in a specific manner to prevent their exonucleolytic expansion

**Figure 4.**
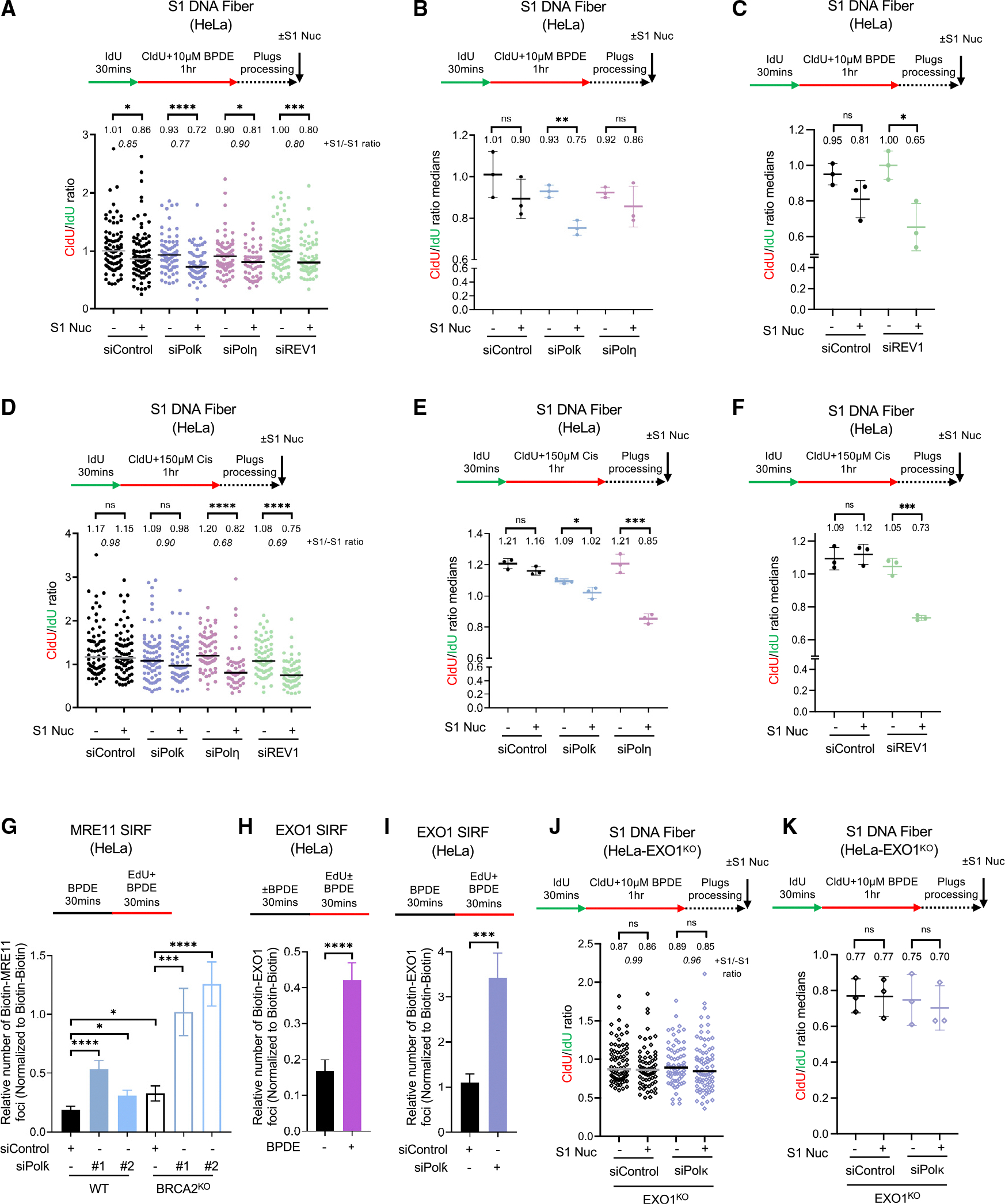
TLS polymerases suppress nascent strand ssDNA gap accumulation in an adduct-specific manner (A–F) S1 nuclease DNA fiber combing assays showing that Polκ knockdown specifically increases nascent strand ssDNA gap formation upon BPDE exposure (A–C) and Polη knockdown specifically increases nascent strand ssDNA gap formation upon cisplatin exposure (D–F) in HeLa cells. REV1 depletion increases nascent strand ssDNA gaps upon both BPDE and cisplatin exposure (A, C, D, and F). (A and D) The ratio of CldU to IdU tract lengths from a representative experiment is presented, with the median values marked on the graphs and listed at the top. At least 55 tracts were quantified for each sample. Asterisks indicate statistical significance (Mann-Whitney, two-tailed). (B, C, E, and F) Quantification of median CldU/IdU ratios from three independent experiments. The mean values and standard deviations are shown. Asterisks indicate statistical significance (t test, two-tailed, unpaired). The data for the other two experiments are presented in Table S1. Schematic representations of the assay conditions are shown at the top. (G–I) SIRF experiments showing that the recruitment of nucleases MRE11 (G) and EXO1 (H and I) to nascent DNA upon BPDE exposure in HeLa cells is increased by Polκ knockdown. At least 68 cells were quantified for each condition. Bars indicate the mean values, error bars represent standard errors of the mean, and asterisks indicate statistical significance (t test, two-tailed, unpaired). Schematic representations of the assay conditions are shown at the top. (J and K) S1 nuclease DNA fiber combing assay showing that EXO1 deletion suppresses ssDNA gap accumulation in Polκ-depleted HeLa cells. (J) The ratio of CldU to IdU tract lengths is presented, with the median values marked on the graphs and listed at the top. At least 65 tracts were quantified for each sample. Asterisks indicate statistical significance (Mann-Whitney, two-tailed). (K) Quantification of median CldU/IdU ratios from three independent experiments. The mean values and standard deviations are shown. Asterisks indicate statistical significance (t test, two-tailed, unpaired). The data for the other two experiments are presented in Table S1. Schematic representations of the assay conditions are shown at the top. See also Figure S4 and Table S1.

Restart of stalled replication forks by PrimPol leaves behind ssDNA gaps. Accumulation of these gaps has recently emerged as a possible determinant of cellular sensitivity to DNA-damaging agents, particularly in BRCA-deficient cells.^20,37–41^ The results described above indicated that TLS polymerases are recruited to DNA adducts in a PrimPol-dependent manner. We thus decided to investigate if this recruitment is physiologically relevant for the repair of PrimPol-generated ssDNA gaps. To address this question, we employed the S1 nuclease DNA fiber combing assay to measure ssDNA gap accumulation. The S1 nuclease will cleave ssDNA that was created during the CldU and BPDE co-treatment, leading to a decreased CldU/IdU ratio. Treating wild-type HeLa cells with 10 μM BPDE resulted in a slight decrease of CldU/IdU ratios in the S1-treated samples compared to the non-S1-treated samples, indicating a mild accumulation of ssDNA gaps. In contrast, the depletion of Polκ led to a statistically significant decrease in the CldU/IdU ratio, as measured by analyzing the median CldU/IdU ratio values from three independent experiments (Figures 4A–4C). These findings indicate that Polκ is necessary to suppress BPDE-induced ssDNA gap accumulation. In contrast, Polη depletion did not affect gap accumulation to a similar extent. On the other hand, when we measured cisplatin-induced ssDNA gap accumulation, we observed that Polη depletion had a major, statistically significant impact on ssDNA gap accumulation, while Polκ depletion only showed a minor effect (Figures 4D–4F). As expected, S1 treatment did not change the length of the IdU tracts (Figure S4A), indicating that the gaps are formed upon repriming on the nascent strand rather than on previously synthesized DNA. Overall, these findings indicate that TLS polymerases are required for the filling of adduct-induced ssDNA gaps in an adduct-specific manner, even in BRCA-proficient cells.

We next investigated the impact of extension TLS polymerases. Depletion of REV1 or REV3 in wild-type HeLa cells resulted in an increase in ssDNA accumulation in response to BPDE or cisplatin, similar to the effect observed upon depletion of the respective bypass polymerase (Figures 4A–4F and S4B). These findings argue that extension by the REV1-Polζ complex upon initial bypass by Polκ or Polη is a critical step in gap filling.

We recently showed that, in BRCA-deficient cells, ssDNA gaps induced by cisplatin are expanded bidirectionally by the exonuclease activities of EXO1 (in the 5′-3′ direction) and MRE11 (in the 3′-5′ direction).^42^ Using SIRF assays designed to take into account the directionality of these exonucleases as we previously reported,^42,43^ we found that both MRE11 and EXO1 are recruited to nascent DNA upon treatment with BPDE in both wild-type and BRCA2-knockout cells (Figures 4G–4I, S2L, and S2M). Depletion of Polκ resulted in the increased recruitment of MRE11 and EXO1 under these conditions, suggesting that they are involved in expanding BPDE-induced gaps in the absence of Polκ-mediated bypass of BPDE adducts. Indeed, S1 nuclease DNA fiber combing assays showed that the loss of EXO1 suppressed BPDE-induced ssDNA gap accumulation in Polκ-depleted cells (Figures 4J and 4K). Overall, these findings indicate that, in the absence of TLS polymerase-mediated gap filling, adduct-induced gaps are exonucleolytically expanded.

### Nucleolytic processing of adduct-induced ssDNA gaps underlies their cytotoxicity

**Figure 5.**
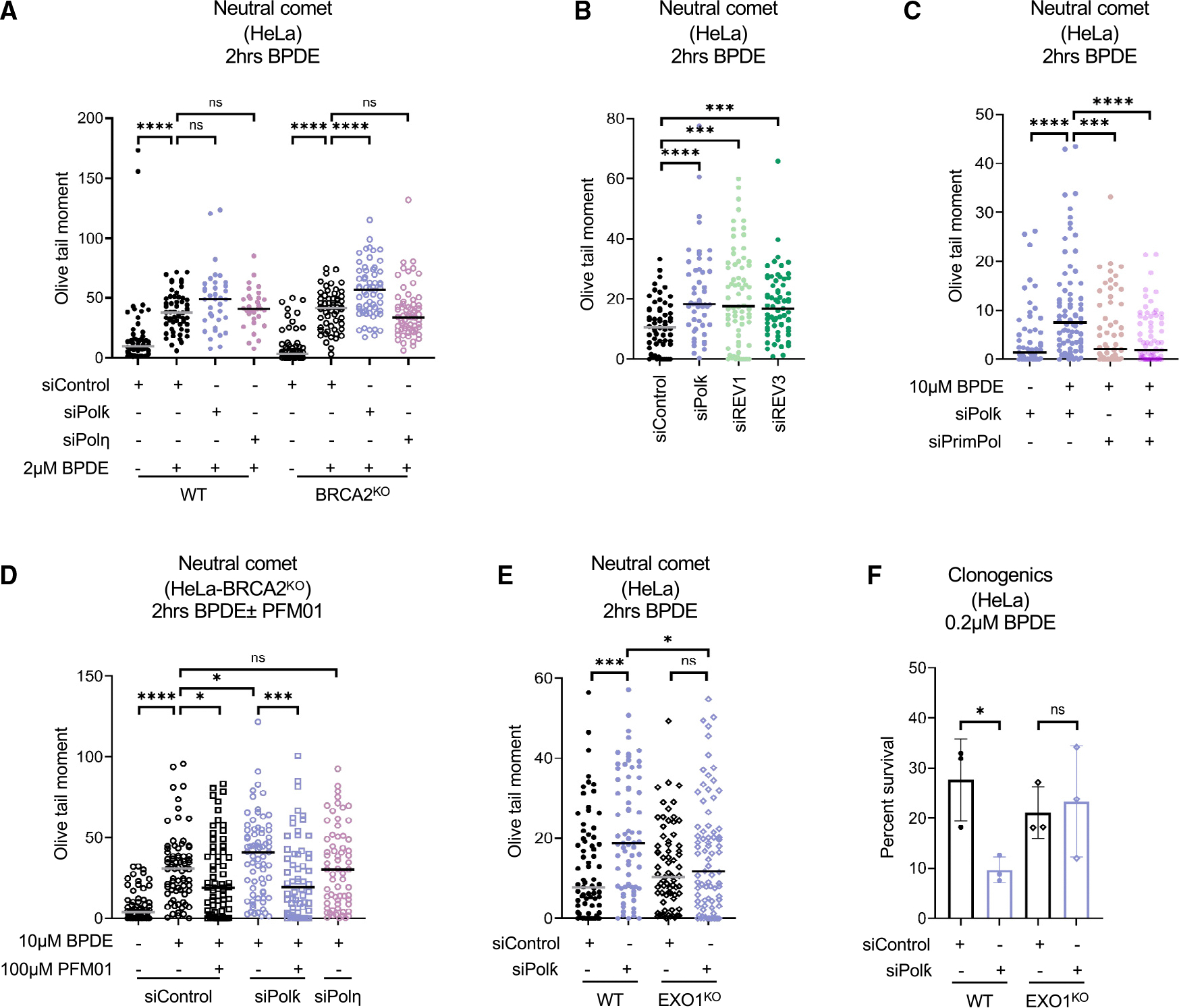
Defects in ssDNA gap filling result in double-strand breaks and cellular sensitivity due to nucleolytic processing of gaps (A) Neutral comet assay showing that BPDE exposure causes DSBs in HeLa cells, which are specifically increased by Polκ depletion. At least 30 comets were quantified for each sample. The median values are marked on the graph, and asterisks indicate statistical significance (Mann-Whitney, two-tailed). (B) Neutral comet assay showing that depletion of REV1 and REV3 increases DSB formation upon BPDE exposure in HeLa cells. At least 50 comets were quantified for each sample. The median values are marked on the graph, and asterisks indicate statistical significance (Mann-Whitney, two-tailed). (C) Neutral comet assay showing that co-depletion of PrimPol suppresses the increase in DSB formation upon Polκ knockdown in BPDE-treated HeLa cells. At least 55 comets were quantified for each sample. The median values are marked on the graph, and asterisks indicate statistical significance (Mann-Whitney, two-tailed). Western blots showing the co-depletion are presented in Figure S1H. (D) Neutral comet assay showing that treatment with the MRE11 endonuclease inhibitor PFM01 suppresses the increase in DSB formation upon Polκ knockdown in BPDE-treated HeLa cells. At least 60 comets were quantified for each sample. The median values are marked on the graph, and asterisks indicate statistical significance (Mann-Whitney, two-tailed). (E) Neutral comet assay showing that deletion of EXO1 suppresses the increase in DSB formation upon Polκ knockdown in BPDE-treated HeLa cells. At least 70 comets were quantified for each sample. The median values are marked on the graph, and asterisks indicate statistical significance (Mann-Whitney, two-tailed). (F) Cellular viability assays showing that deletion of EXO1 suppresses the BPDE sensitivity of Polκ-depleted HeLa cells. The average of three independent experiments, with standard deviations indicated as error bars, is shown. Asterisks indicate statistical significance (two-way ANOVA). See also Figures S1 and S5 and Table S1.

Finally, we sought to investigate how TLS-mediated filling of adduct-induced ssDNA gaps affects genomic stability. We first measured the formation of DSBs using the neutral comet assay. Treating HeLa cells with 2 μM BPDE for 2 h resulted in an increase in DSBs, which was accentuated by the depletion of Polκ, but not Polη, with a more drastic effect observed in BRCA2-knockout cells (Figures 5A and S5A). These findings are in line with a previous study employing γH2AX phosphorylation levels as a marker of DNA damage.^15^ A larger increase upon Polκ depletion was observed when increasing the BPDE treatment dose to 10 μM (Figures 5B and S5B). Moreover, depletion of REV1 or REV3 caused a similar increase in DSB formation under these conditions. These findings indicate that bypass and extension TLS polymerases are required to suppress DSB formation induced by BPDE adduct formation.

We next investigated the mechanism of DSB formation under these conditions. To differentiate between DSBs potentially formed at arrested but not restarted forks and those formed on ssDNA gaps resulting from PrimPol-mediated restart, we co-depleted PrimPol. We observed that PrimPol depletion suppresses BPDE-induced DSB formation in Polκ-knockdown cells (Figures 5C, S1H, and S5C). These findings indicate that BPDE-induced DSB accumulation in the absence of adduct bypass requires ssDNA gap formation by PrimPol-mediated repriming.

We previously showed that exonucleolytic expansion of cisplatin-induced gaps in BRCA2-knockout cells promotes their conversion into DSBs, which occurs through the subsequent endonucleolytic activity of the MRE11 endonuclease on the parental strand at the ssDNA gap region.^42,43^ In line with this mechanism, loss of EXO1 or inhibition of the MRE11 endonuclease activity using the specific inhibitor PFM01 also suppressed BPDE-induced DSB formation in Polκ-depleted cells (Figures 5D, 5E, and S5D–S5F). PFM01 treatment also suppressed cisplatin-induced DSB formation in Polη-depleted cells (Figure S5G). Overall, these findings indicate that, in the absence of lesion bypass, ssDNA gaps cannot be filled and are instead nucleolytically processed into DSBs.

The loss of Polκ was previously shown to result in cellular sensitivity to BPDE treatment.^15^ We thus investigated if this sensitivity reflects the role of Polκ in suppressing the nucleolytic processing of ssDNA gaps. In line with these previously published findings, we observed that Polκ depletion sensitized HeLa cells to BPDE. However, the loss of EXO1 suppressed this sensitization (Figure 5F). This indicates that the nucleolytic processing of BPDE-induced ssDNA gaps underlies their cytotoxicity, and Polκ promotes BPDE resistance by filling BPDE-induced ssDNA gaps.

## DISCUSSION

**Figure 6.**
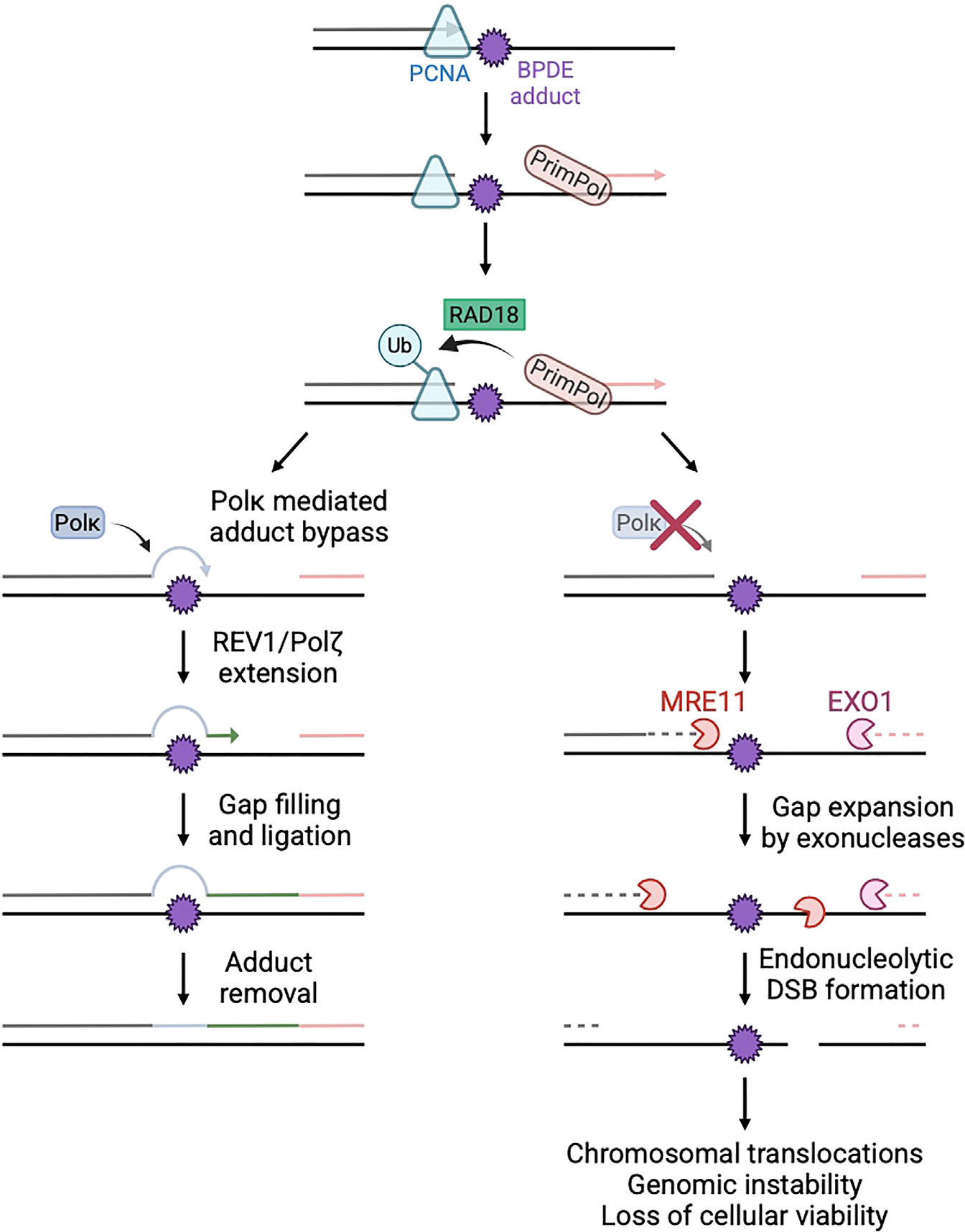
Schematic representation of the proposed models PCNA ubiquitination and subsequent TLS polymerase recruitment occur only upon fork restart by PrimPol. Once the appropriate polymerase is recruited in an adduct-specific manner (shown here for Polκ and BPDE), the extension complex REV1-Polζ fills the gap. In the absence of the adduct-specific bypass polymerase or of the extension polymerases, ssDNA gaps are processed into DSBs by the nucleases EXO1 and MRE11. This processing causes the cellular sensitivity to the adduct. Created in BioRender (https://BioRender.com/p58a011).

Our findings indicate that TLS polymerases mainly act on ssDNA gaps formed upon PrimPol-mediated fork restart to bypass the adducts in an adduct-specific manner and therefore fill the gap. In the absence of the specific TLS polymerase required for adduct bypass and gap filling, the gaps are exonucleolytically expanded and subsequently converted into DSBs (Figure 6). Importantly, we show that this nucleolytic conversion underlies the cytotoxicity of adduct-inducing DNA-damaging agents.

Previous work in yeast has shown that restricting PCNA ubiquitination to G2 is enough to confer DNA damage tolerance, indicating that TLS can take place after DNA replication.^44^ However, whether post-replication TLS occurs under normal conditions in higher eukaryotes was still unclear. Thus, whether TLS occurs on the fly to restart stalled replication forks without discontinuities in the nascent strand or instead acts behind restarted replication forks to fill up such discontinuities is a major open question. The fact that, in our study, PrimPol depletion led to a reduction in PCNA ubiquitination at nascent DNA upon BPDE or cisplatin treatment to near-background levels, coupled with a similarly reduced recruitment of TLS polymerases under these conditions, suggests that TLS occurs primarily on ssDNA gaps left behind PrimPol-restarted forks. These results are in line with the S1 nuclease DNA fiber combing results showing that the loss of TLS polymerases causes an accumulation of ssDNA gaps in an adduct-specific manner. Overall, our results imply that, at least in the case of cisplatin and BPDE adducts, TLS is not involved in restarting forks on the fly.

These findings have potential implications for both carcinogenesis and chemotherapy. While exposure to BPDE has been documented to cause point mutations, our work implies that if gap filling is not efficient, then these gaps are transformed into DSBs, which may initiate chromosomal translocations. Thus, structural chromosome changes may represent an unappreciated type of genomic instability upon BPDE exposure since chromosomal translocations are more difficult to determine from current next-generation sequencing approaches, which only generate short reads. This type of genomic instability may potentially be contributing to BPDE-induced carcinogenesis.

Our work also sheds light on the poorly defined concept of fork collapse, which refers to the generation of DSBs at arrested forks.^45^ We find that BPDE-induced DSBs occur in a PrimPol-, EXO1-, and MRE11-dependent manner. This suggests that, at least under the conditions investigated here, the forks do not get cleaved upon prolonged arrest, but rather their PrimPol-mediated restart causes the formation of unstable ssDNA gaps, which represent the structure that gets cleaved to form DSBs. If true, then this model could have implications for both developing preventive approaches to mitigate adduct-induced carcinogenesis and improving treatments with genotoxic chemotherapies.

### Limitations of the study

While the SIRF results presented here show statistically significant differences, for some of the proteins investigated, the differences observed (changes induced by DNA damage exposure or PrimPol depletion) are not large. This likely reflects the high background of the SIRF experimental setup, as the SIRF signal observed without DNA damage exposure or upon PrimPol depletion is similar to what is observed upon depletion of the specific protein investigated. Conceptually, the finding that EXO1 depletion suppresses ssDNA gap accumulation may reflect not only a role of EXO1 in gap expansion as discussed above, which would antagonize gap filling, but also the possibility that EXO1 activity is necessary for gap formation in the first place. Finally, since *in vitro* REV1 is also able to insert nucleotides across BPDE lesions,^46^ the observed role of REV1 in gap suppression may reflect its lesion bypass activity rather than extension DNA synthesis after Polκ bypass.

## RESOURCE AVAILABILITY

### Lead contact

Further information and requests for resources and reagents should be directed to and will be fulfilled by the lead contact, Claudia M. Nicolae (cmn14@psu.edu).

### Materials availability

This study did not generate unique reagents.

### Data and code availability

- All data supporting the findings of this study are available within the article and its supplemental information. The source data underlying each of the main and supplemental figures, including the values plotted in graphs, the exact *p* values, and the uncropped western blot images, are presented in Table S1.
- This paper does not report original code.
- Any additional information required to reanalyze the data reported in this paper is available from the lead contact upon request.

## STAR★METHODS

### EXPERIMENTAL MODEL AND STUDY PARTICIPANT DETAILS

#### Cell lines

HeLa cervical carcinoma cells derived from a 31-year-old female patient were obtained authenticated from ATCC (CCL-2). Cells were grown in Dulbecco’s modified Eagle’s media (DMEM). HeLa-BRCA2^KO47^ and HeLa-EXO1^KO42^ cells were generated in our laboratory and previously described. Cells were tested for mycoplasma and confirmed negative.

### METHOD DETAILS

#### Cell and protein techniques

Gene knockdown was performed using Lipofectamine RNAiMAX. Denatured whole cell extracts were prepared by boiling cells in 100mM Tris, 4% SDS, 0.5M β-mercaptoethanol.

#### Comet assays

Neutral comet assays were performed using the Comet Assay Kit (Trevigen, 4250–050). Chemical compounds were added according to the labeling schemes presented. Slides were imaged on a Nikon microscope operating the NIS Elements V1.10.00 software. Olive tail moment was analyzed using CometScore 2.0.

#### Drug sensitivity assays

To assess cellular viability upon drug treatment, a luminescent ATP-based assay was performed using the CellTiterGlo reagent (Promega G7572) according to the manufacturer’s instructions. Following treatment with siRNA, 1500 cells were seeded per well in 96-well plates and incubated as indicated for 3 days. Luminescence was quantified using a Promega GloMax Navigator plate reader.

#### DNA fiber combing assays

Cells were incubated with 100μM IdU and 100μM CldU as indicated. Chemical compounds (BPDE, cisplatin) were added according to the labeling schemes presented. Next, cells were collected and processed using the FiberPrep kit (Genomic Vision EXT-001) according to the manufacturer’s instructions. Samples were added to combing reservoirs containing MES solution (2-(N-morpholino) ethanesulfonic acid) and DNA molecules were stretched onto coverslips (Genomic Vision COV-002-RUO) using the FiberComb Molecular Combing instrument (Genomic Vision MCS-001). For S1 nuclease assays, MES solution was supplemented with 1mM zinc acetate and either 40U/mL S1 nuclease (ThermoFisher 18001016) or S1 nuclease dilution buffer as control, and incubated for 30 minutes at room temperature. Slides were then stained with antibodies detecting CldU (Abcam 6326) and IdU (BD 347580), and incubated with secondary Cy3 (Abcam 6946) or Cy5 (Abcam 6565) conjugated antibodies. Finally, the cells were mounted onto coverslips and imaged using a confocal microscope (Leica SP5) and analyzed using LASX 3.5.7.23225 software.

#### Proximity ligation-based assays

For proximity ligation assays, cells were seeded into 8-chamber slides and 24 hours later, were treated with chemical compounds (BPDE, cisplatin) as indicated. Cells were then permeabilized with 0.5% Triton for 10min at 4°C, washed with PBS, fixed at room temperature with 3% paraformaldehyde in PBS for 10min, washed again in PBS and then blocked in Duolink blocking solution (Millipore Sigma DUO82007) for 1hr at 37°C, and incubated overnight at 4°C with primary antibodies. Antibodies used were: Polκ (Bethyl A301–977A), Polη (Cell Signaling Technology 13848) and BPDE-DNA (Santa Cruz Biotechnology sc-52624). Samples were then subjected to a proximity ligation reaction using the Duolink kit (Millipore Sigma DUO92008) according to the manufacturer’s instructions. Slides were imaged using a Deltavision microscope with SoftWorx 6.5.2 software, and images were analyzed using ImageJ 1.53a software.

For SIRF assays, cells were seeded into 8-chamber slides and 24 hours later they were pulse-labeled with 50μM EdU and treated with chemical compounds (BPDE, cisplatin) according to the labeling schemes presented. Cells were permeabilized with 0.5% Triton for 10min at 4°C, washed with PBS, fixed at room temperature with 3% paraformaldehyde in PBS for 10min, washed again in PBS, and then blocked in 3% BSA in PBS for 30min. Cells were then subjected to Click-iT reaction with biotin-azide using the Click-iT Cell Reaction Buffer Kit (ThermoFisher C10269) for 30min and incubated overnight at 4°C with primary antibodies diluted in PBS with 1% BSA. The primary antibodies used were: Biotin (mouse: Jackson ImmunoResearch 200–002-211; rabbit: Bethyl Laboratories A150–109A); Polκ (Santa Cruz Biotechnology sc-166667), Polη (Santa Cruz Biotechnology sc-17770), Ubiquityl-PCNA Lys164 (Cell Signaling Technology 13439); REV1 (Santa Cruz Biotechnology sc-393022), REV3 (GeneTex GTX100153); MRE11 (GeneTex GTX70212); EXO1 (Santa Cruz Biotechnology sc-56092); PrimPol (Proteintech 29824–1-AP). Next, samples were subjected to a proximity ligation reaction using the Duolink kit (MilliporeSigma DUO92008) according to the manufacturer’s instructions. Slides were imaged using a Deltavision microscope with SoftWorx 6.5.2 software, and images were analyzed using ImageJ 1.53a software. To account for variation in EdU uptake between samples, for each sample, the number of protein-biotin foci were normalized to the average number of biotin-biotin foci for that respective sample. The scale bars for the SIRF and PLA micrographs shown represent 10μm.

### QUANTIFICATION AND STATISTICAL ANALYSIS

For SIRF and PLA assays the t test (two-tailed, unpaired) was used. For DNA fiber assays and comet assays the Mann-Whitney statistical test (two-tailed) was performed. For CellTiterGlo cellular viability assays the t test (two-tailed, unpaired) was used. In general, results from one experiment are shown; the results were reproduced in at least one additional biological conceptual replicate. Statistical analyses were performed using GraphPad Prism 10 and Microsoft Excel v2205 software. Statistical significance is indicated for each graph (ns = not significant, for *p* > 0.05; * for *p* ≤ 0.05; ** for *p* ≤ 0.01; *** for *p* ≤ 0.001, **** for *p* ≤ 0.0001). The source data underlying all figure are provided in Table S1.

## Supplementary Material

1

2

## Figures and Tables

**KEY RESOURCES TABLE:** 

REAGENT or RESOURCE	SOURCE	IDENTIFIER
Antibodies		
Polκ	Santa Cruz Biotechnology	sc-166667; RRID:AB_2167029
Polη	Cell Signaling Technology	13848; RRID:AB_2798329
PrimPol	Proteintech	29824-1-AP
RAD18	Cell Signaling Technology	9040; RRID:AB_2918349
USP1	Abcam	ab264221;
REV3	GeneTex	GTX100153; RRID:AB_2037856
UBC13	Santa Cruz Biotechnology	sc-376470; RRID:AB_11150503
Vinculin	Santa Cruz Biotechnology	sc-73614; RRID:AB_1131294
GAPDH	Santa Cruz Biotechnology	sc-47724; RRID:AB_627678
CldU	Abcam	ab6326
IdU	BD	347580; RRID:AB_10015219
Cy3	Abcam	ab6946; RRID:AB_955045
Cy5	Abcam	ab6565; RRID:AB_955063
Polκ	Bethyl	A301-977A; RRID:AB_1548020
BPDE-DNA	Santa Cruz Biotechnology	sc-52624; RRID:AB_628803
Biotin (mouse)	Jackson ImmunoResearch;	200-002-211; RRID:AB_2339006
Biotin (rabbit)	Bethyl	A150-109A; RRID:AB_67327
Polη	Santa Cruz Biotechnology	sc-17770; RRID:AB_2167007
Ubiquityl-PCNA Lys164	Cell Signaling Technology	13439; RRID:AB_2798219
REV1	Santa Cruz Biotechnology	sc-393022; RRID:AB_2885169
EXO1	Santa Cruz Biotechnology	sc-56092; RRID:AB_783300
MRE11	GeneTex	GTX70212; RRID:AB_372398
Chemicals, peptides, and recombinant proteins		
PFM01	Tocris	6222
BPDE	Penn State Cancer Institute Organic Synthesis Core Facility	N/A
S1 nuclease	ThermoFisher	18001016
Critical commercial assays		
Comet Assay Kit	Trevigen	4250-050
CellTiterGlo reagent	Promega	G7572
FiberPrep kit	Genomic Vision	EXT-001
Duolink kit	Millipore Sigma	DUO92008
Click-iT Cell Reaction Buffer Kit	ThermoFisher	C10269
Lipofectamine RNAiMAX	ThermoFisher	13778500
Duolink blocking solution	Millipore Sigma	DUO82007
Deposited data		
Source data		Table S1
Experimental models: Cell lines		
HeLa	ATCC	CCL-2
Oligonucleotides		
AllStars Negative Control siRNA	Qiagen	1027281
Polκ	ThermoFisher	Assay ID 117964
Polη	ThermoFisher	Assay ID 119730
PrimPol^#1^	ThermoFisher	Assay ID 39536
PrimPol^#2^	ThermoFisher	Assay IDs47418
RAD18	ThermoFisher	Assay ID s32295
USP1	ThermoFisher	Assay IDs14724
REV1	ThermoFisher	GAAAUCCUUGCAGAGACCAAACUUA
REV3	ThermoFisher	Assay ID 12291
UBC13	ThermoFisher	Assay ID: s14595
Software and algorithms		
SoftWorx 6.5.2	Cytiva	N/A
LASX 3.5.7.23225	Leica	N/A
NIS Elements V1.10.00	Nikon	N/A
CometScore 2.0	RexHoover.com	N/A
ImageJ 1.53a	NIH	N/A

## Highlights

- Polκ is recruited to BPDE lesions and Polη to cisplatin lesions during DNA replication
- TLS polymerase recruitment requires PrimPol-mediated fork restart
- TLS polymerase deficiency results in ssDNA gap accumulation in an adduct-specific manner
- These gaps are processed by nucleases, resulting in cytotoxic double-stranded DNA breaks
