# Supplementary material for: Translesion-synthesis-mediated bypass of DNA lesions occurs predominantly behind replication forks restarted by PrimPol
Source: Cell Rep. Author manuscript; Available in PMC 2025 May 5. (PMC12051500; doi:10.1016/j.celrep.2025.115360)
Supplement: 1 [file NIHMS2069113-supplement-1.pdf]

Cell Reports, Volume *44*

Supplemental information

Translesion-synthesis-mediated bypass of DNA lesions occurs predominantly behind replication forks restarted by PrimPol

Ashna Dhoonmoon, Julia R. Ambrose, Sonal Garg, Cynthia Lascarez-Espana, Abbey Rebok, Thomas E. Spratt, George-Lucian Moldovan, and Claudia M. Nicolae

Figure S1

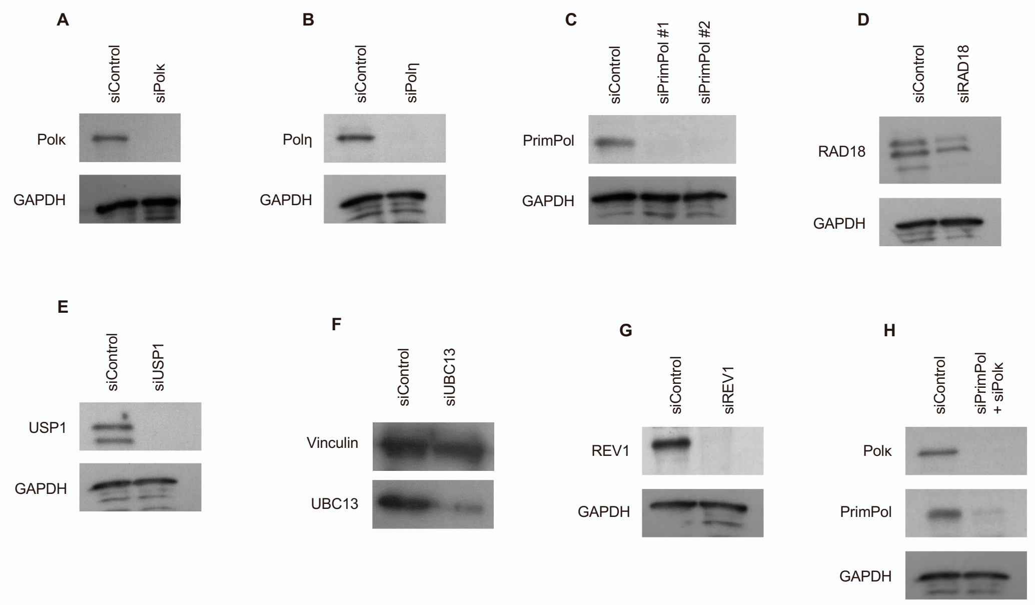

Figure S1. Confirmation of gene knockdowns and knockouts, related to Figures 1, 2, 3 and 5.

Western blots showing the siRNA-mediated knockdown of Polκ (**A**), Polη (**B**), PrimPol (**C**), RAD18 (**D**), USP1 (**E**), UBC13 (**F**), REV1 (**G**), and PRIMPOL-Polκ co-depletion (**H**) in HeLa cells.

Figure S2

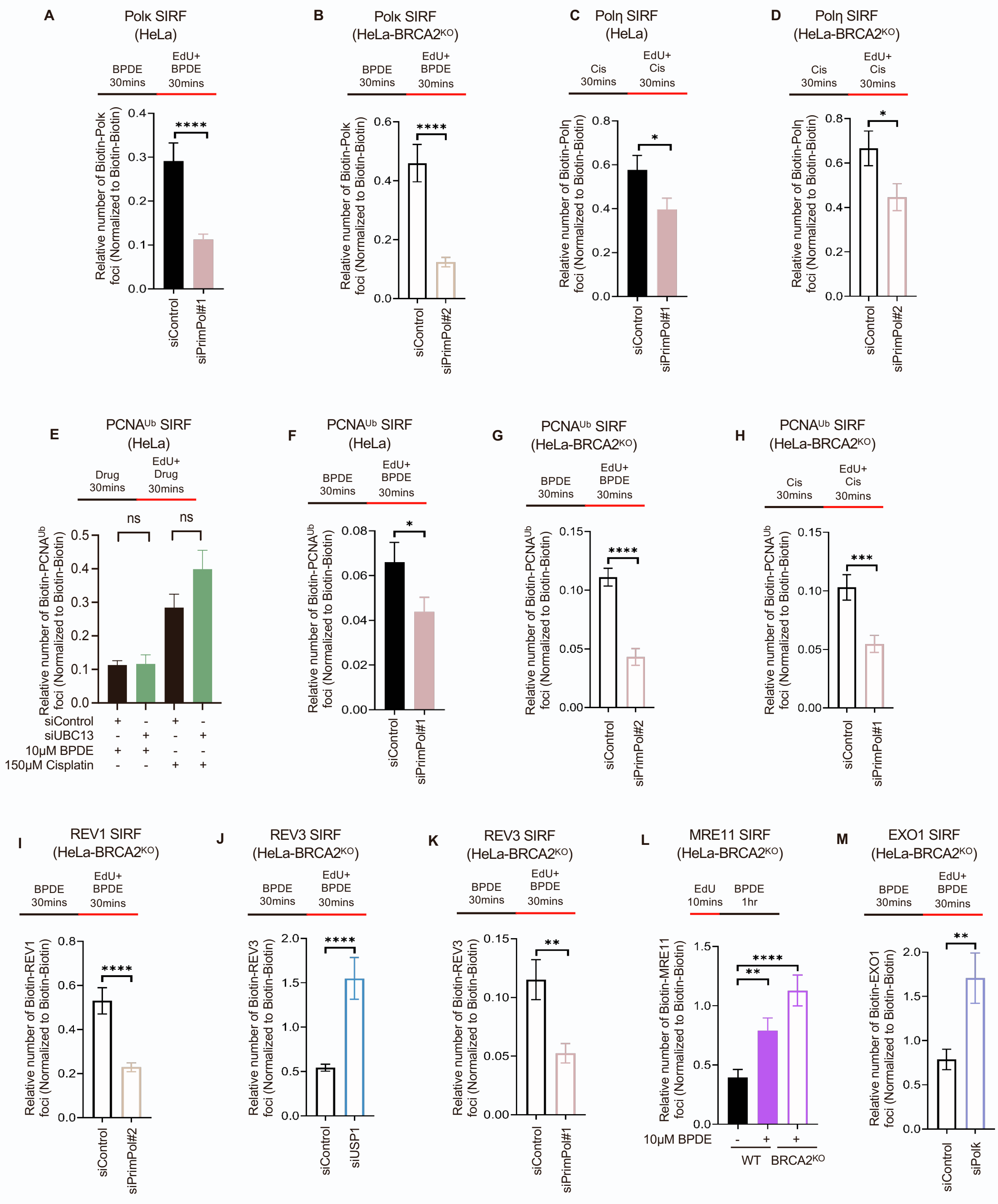

A
Polκ SIRF (HeLa)
BPDE 30mins
EdU+ BPDE 30mins
Relative number of Biotin-Polκ foci (Normalized to Biotin-Biotin)
****
siControl
siPrimPol#1
B
Polκ SIRF (HeLa-BRCA2KO)
siPrimPol#2
C
Polη SIRF (HeLa)
Cis 30mins
EdU+ Cis 30mins
Relative number of Biotin-Polη foci (Normalized to Biotin-Biotin)
D
Polη SIRF (HeLa-BRCA2KO)
E
PCNAUb SIRF (HeLa)
Drug 30mins
EdU+ Drug 30mins
ns
Relative number of Biotin-PCNAUb foci (Normalized to Biotin-Biotin)
siControl
siUBC13
10μM BPDE
150μM Cisplatin
F
PCNAUb SIRF (HeLa)
G
PCNAUb SIRF (HeLa-BRCA2KO)
H
PCNAUb SIRF (HeLa-BRCA2KO)
I
REV1 SIRF (HeLa-BRCA2KO)
Relative number of Biotin-REV1 foci (Normalized to Biotin-Biotin)
J
REV3 SIRF (HeLa-BRCA2KO)
Relative number of Biotin-REV3 foci (Normalized to Biotin-Biotin)
siUSP1
K
REV3 SIRF (HeLa-BRCA2KO)
L
MRE11 SIRF (HeLa-BRCA2KO)
EdU 10mins
BPDE 1hr
Relative number of Biotin-MRE11 foci (Normalized to Biotin-Biotin)
10μM BPDE
WT
BRCA2KO
M
EXO1 SIRF (HeLa-BRCA2KO)
Relative number of Biotin-EXO1 foci (Normalized to Biotin-Biotin)
siPolk

Figure S2. SIRF assays, related to Figures 2 and 3.

(A-D) SIRF assays showing that depletion of PRIMPOL suppresses the recruitment of TLS polymerases Polκ (**A,B**) and Polη (**C,D**) to nascent DNA upon adduct formation in HeLa cells. At least 75 cells were quantified for each condition.

(E-H) SIRF experiments showing the impact of UBC13 (**E**) or PrimPol (**F-H**) depletion on PCNA ubiquitination on nascent DNA upon adduct formation. At least 75 cells were quantified for each condition.

(I-K) SIRF experiments showing the impact of PrimPol depletion on the recruitment of TLS polymerases REV1 (**I**) and REV3 (**J,K**) to nascent DNA upon adduct formation in HeLa cells. At least 75 cells were quantified for each condition.

(L,M) SIRF experiments showing the impact the recruitment of nucleases MRE11 (**L**) and EXO1 (**M**) to nascent DNA upon adduct formation in HeLa cells. At least 75 cells were quantified for each condition.

Bars indicate the mean values, error bars represent standard errors of the mean, and asterisks indicate statistical significance (t-test, two-tailed, unpaired). Schematic representations of the assay conditions are shown at the top.

Figure S3

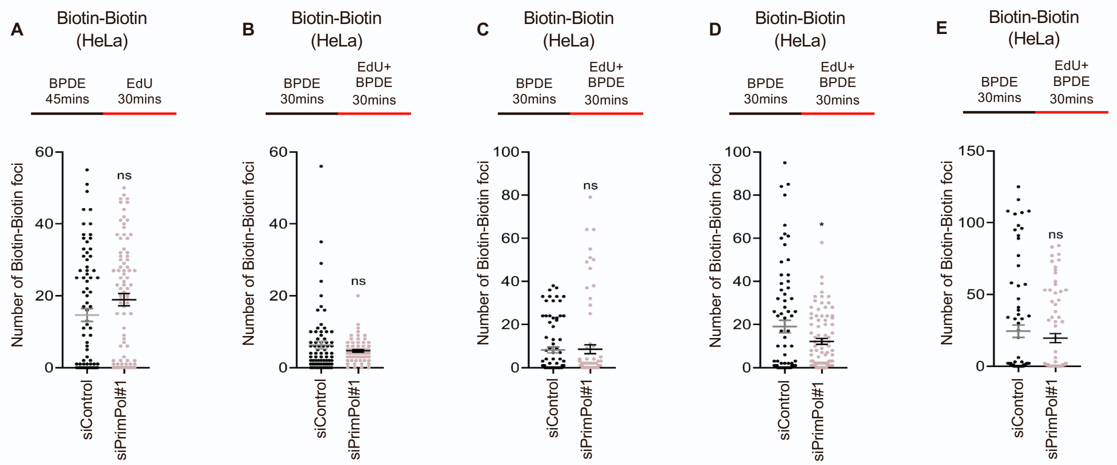

Figure S3. Control SIRF assays, related to Figures 2 and 3.

PrimPol depletion does not affect EdU incorporation under the BPDE treatment conditions investigated. Quantifications of biotin-biotin foci are presented, corresponding to the experiments presented in Figure 2A (**A**), 2B (**B**), 2H (**C**), 3B (**D**) and S2K (**E**).

Figure S4

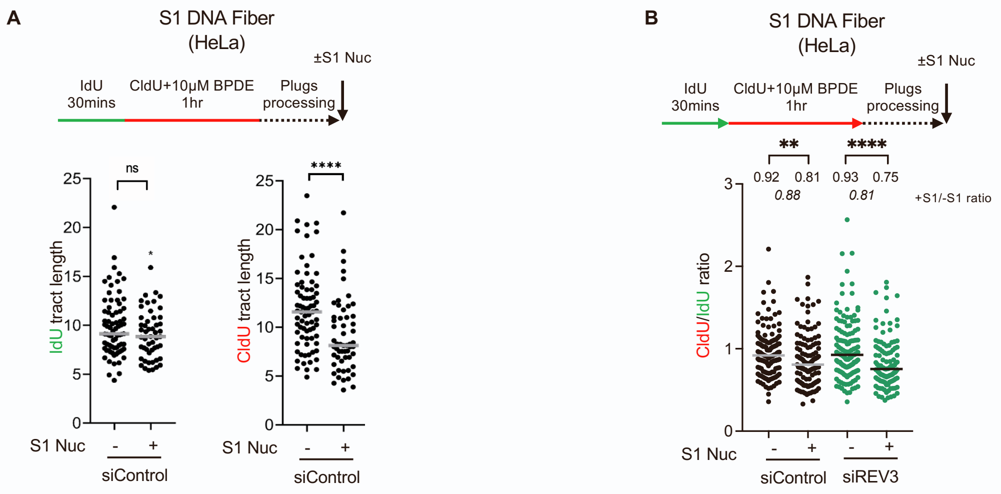

Figure S4. S1 nuclease DNA fiber combing assays, related to Figure 4.

(A) S1 nuclease DNA fiber combing assays showing that BPDE treatment does not induce ssDNA gaps on previously synthesized DNA. Quantification of the IdU tract is presented, with the median values marked on the graphs and listed at the top. At least 50 tracts were quantified for each sample. Asterisks indicate statistical significance (Mann-Whitney, two-tailed). A schematic representation of the assay conditions is shown at the top.

(B) S1 nuclease DNA fiber combing assays showing that REV3 knockdown increases nascent strand ssDNA gap formation upon BPDE exposure in HeLa cells. The ratio of CldU to IdU tract lengths is presented, with the median values marked on the graphs and listed at the top. At least 115 tracts were quantified for each sample (pooled from two independent experiments). Asterisks indicate statistical significance (Mann-Whitney, two-tailed). A schematic representation of the assay conditions is shown at the top.

Figure S5

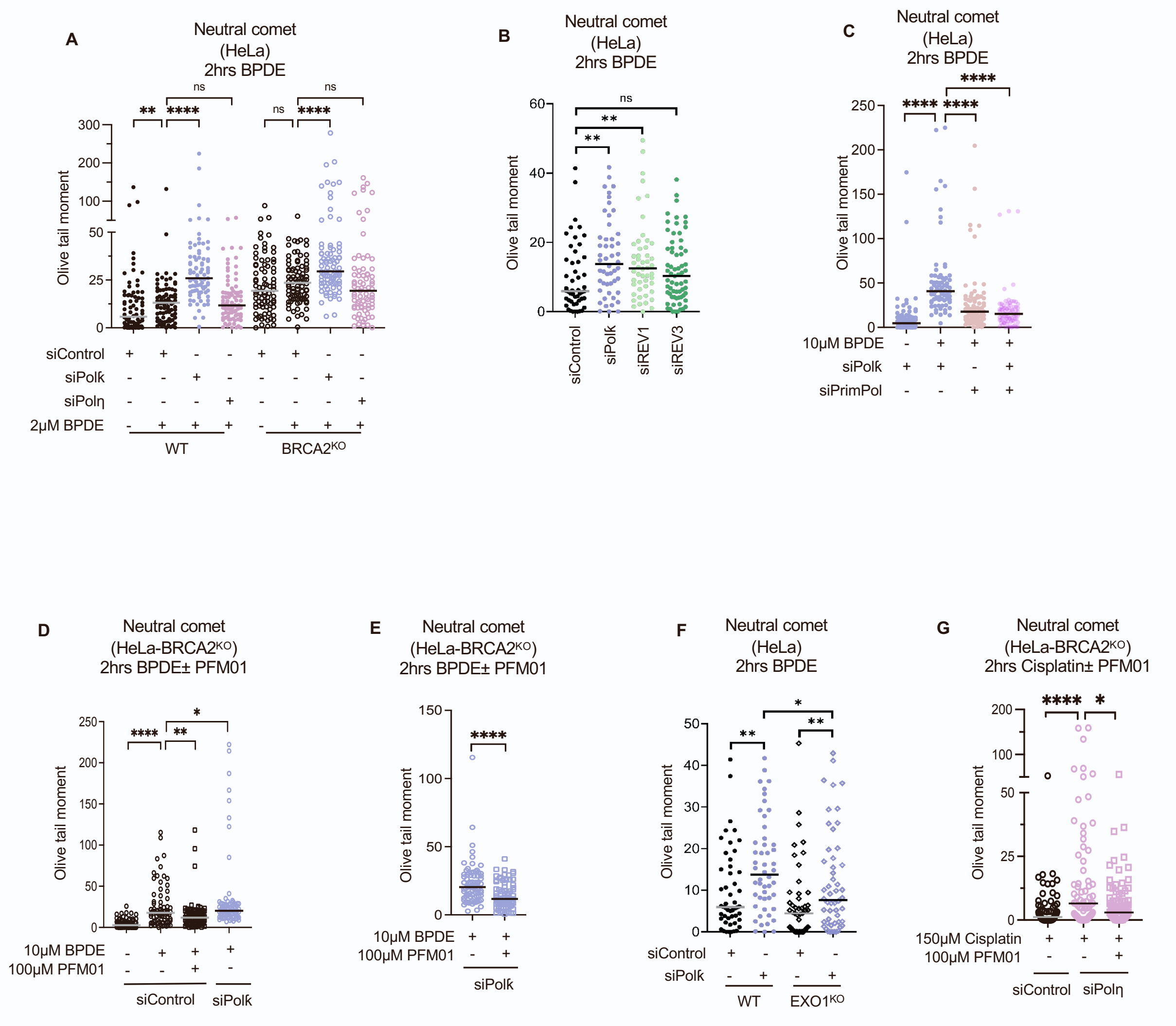

Figure S5. Independent biological replicate experiments of the neutral comet assays, related to Figure 5.

(A) Neutral comet assay showing that BPDE exposure causes DSBs in HeLa cells, which are specifically increased by Polκ depletion. At least 75 comets were quantified for each sample. The median values are marked on the graph, and asterisks indicate statistical significance (Mann-Whitney, two-tailed).

(B) Neutral comet assay showing that depletion of REV1 and REV3 increases DSB formation upon BPDE exposure in HeLa cells. At least 50 comets were quantified for each sample. The median values are marked on the graph, and asterisks indicate statistical significance (Mann-Whitney, two-tailed).

(C) Neutral comet assay showing that co-depletion of PRIMPOL suppresses the increase in DSB formation upon Polκ knockdown in BPDE-treated HeLa cells. At least 75 comets were quantified for each sample. The median values are marked on the graph, and asterisks indicate statistical significance (Mann-Whitney, two-tailed).

(D,E) Neutral comet assay showing that treatment with the MRE11 endonuclease inhibitor PFM01 suppresses the increase in DSB formation upon Polκ knockdown in BPDE-treated HeLa cells. At least 75 comets were quantified for each sample. The median values are marked on the graph, and asterisks indicate statistical significance (Mann-Whitney, two-tailed).

(F) Neutral comet assay showing that deletion of EXO1 suppresses the increase in DSB formation upon Polκ knockdown in BPDE-treated HeLa cells. At least 50 comets were quantified for each sample. The median values are marked on the graph, and asterisks indicate statistical significance (Mann-Whitney, two-tailed).

(G) Neutral comet assay showing that treatment with the MRE11 endonuclease inhibitor PFM01 suppresses the increase in DSB formation upon Polη knockdown in cisplatin-treated HeLa cells. At least 70 comets were quantified for each sample. The median values are marked on the graph, and asterisks indicate statistical significance (Mann-Whitney, two-tailed).
